# Evaluation of Disc-FX versus L’DISQ for percutaneous disc decompression: pilot comparative study using the minimal clinically important difference

**DOI:** 10.1186/s40981-026-00846-8

**Published:** 2026-01-12

**Authors:** Haruka Takaoka, Reon Kobayashi, Ichiro Okano, Asae Taketomi, Eiko Hara, Hitoshi Mera, Masaki Ishikawa, Yoshifumi Kudo, Katsunori Oe

**Affiliations:** 1 Department of Anesthesiology, Showa Medical University School of Medicine, 1-5- 8 Hatanodai, Shinagawa-ku, Tokyo 142-8666 Japan; 2 Department of Orthopedic Surgery, Showa Medical University School of Medicine, 1-5-8 Hatanodai, Shinagawa-ku, Tokyo 142-8666 Japan

**Keywords:** Lumbar disc herniation, Percutaneous disc decompression, Disc-FX, L’DISQ, Minimally invasive spine surgery, Radicular pain, Interventional pain management, Minimal clinically important difference

## Abstract

**Background:**

Lumbar disc herniation often causes disabling radicular pain and impaired quality of life (QOL). Disc-FX and L’DISQ are minimally invasive percutaneous disc decompression devices, but their comparative efficacy remains uncertain.

**Methods:**

We retrospectively reviewed adults with single-level lumbar disc herniation treated with Disc-FX or L’DISQ at a single centre (October 2018–August 2023). Eligibility required radicular pain consistent with magnetic resonance imaging and either positive provocative discography or pain relief after intradiscal injection. Pain intensity (Numerical Rating Scale [NRS]) and QOL (EuroQOL 5-Dimension 5-Levels [EQ-5D-5 L]) were assessed at baseline and up to 6 months. The primary outcome was the between-group difference in change in NRS from baseline to 6 months. Secondary outcomes included 6-month change in EQ-5D-5 L and minimal clinically important difference (MCID) attainment (NRS ≥ 2-point reduction; EQ-5D-5 L ≥ 0.08 increase).

**Results:**

Forty-two patients were included (Disc-FX *n* = 16; L’DISQ *n* = 26). Both procedures reduced NRS pain scores over 6 months, and the primary outcome showed no statistically significant between-group difference (mean difference 1.32 points; 95% CI − 0.36 to 3.01). Among patients with baseline EQ-5D-5 L data (*n* = 38), Disc-FX showed greater 6-month EQ-5D-5 L improvement (mean difference 0.134; 95% CI 0.013 to 0.255) and higher EQ-5D-5 L MCID attainment (83.3% vs. 50.0%), but these are secondary results from a small retrospective cohort.

**Conclusions:**

In this pilot study, Disc-FX and L’DISQ had comparable short-term pain outcomes. Any apparent QOL advantage with Disc-FX should be interpreted cautiously as exploratory and potentially confounded, and requires confirmation in adequately powered prospective comparative studies.

## Background

The lifetime risk of lumbar disc herniation is substantial [1], potentially leading to significant deterioration in quality of life (QOL) due to low back and leg pain [2]. Minimally invasive procedures have been developed to alleviate discogenic and radicular pain by decompressing lumbar disc herniations. These techniques include chymopapain, percutaneous laser disc decompression, automated percutaneous lumbar discectomy, decompression, nucleoplasty, and targeted disc decompression [3]. These techniques provide pain relief comparable to open/microdiscectomy while causing less surgical trauma, reducing pain, shortening surgery time, and enabling quicker recovery and an earlier return to daily activities [3, 4].

Recently, several new devices, such as Disc-FX and L’DISQ, have been developed with multifactorial processes, and good results have been reported. The Disc-FX system combines fluoroscopically guided nucleus pulposus removal, radiofrequency ablation, and annuloplasty [5]. In contrast, L’DISQ is a navigable disc ablation device with a long flexible electrode that enables direct access to disc herniation [6]. Both Disc-FX and L’DISQ are efficacious minimally invasive devices for lumber disc herniations. Studies have confirmed the efficacy and effectiveness of each device [5–7]; however, to the best of our knowledge, there has been no direct comparison of the efficacy of these two devices. Therefore, in this pilot study, we aimed to compare the efficacy of Disc-FX and L’DISQ on pain, QOL improvement, and complication rates in patients with a single intervertebral lumbar disc herniation.

## Methods

### Study population and inclusion/exclusion criteria

This single-centre retrospective cohort included all consecutive patients who underwent Disc-FX or L’DISQ for lumbar disc herniation at our hospital between October 2018 and August 2023. The study was conducted using an opt-out approach, in which eligible patients were informed about the study and could decline participation. The protocol was approved by the Institutional Review Board of Showa Medical University (approval number: 2023-178-B) on May 30, 2024. The study was designed, conducted, and reported in accordance with the Strengthening the Reporting of Observational Studies in Epidemiology (STROBE) guidelines for cohort studies [8].

Inclusion criteria were: (1) single-level lumbar disc herniation on magnetic resonance imaging consistent with radicular symptoms; (2) lumbar radicular pain with either positive provocative discography or pain relief after intradiscal injection; and (3) no prior spinal surgery at the treated level. Exclusion criteria were: (1) traumatic, oncologic, or infectious pathology of the lumbar spine; (2) inability to complete the procedure because of pain; (3) additional percutaneous disc decompression (PDD) for cervical disc herniation; (4) prior spinal surgery within 6 months before PDD; and (5) PDD performed at multiple lumbar levels on the same day.

### Surgical technique

All procedures were performed by two experienced pain specialists under local anesthesia with mild intravenous sedation (pentazocine 15 mg and midazolam 1–2 mg) and prophylactic cefazolin 1 g. Patients were monitored with standard perioperative vital signs and remained awake and able to converse. Under fluoroscopic guidance, the puncture needle was inserted through Kambin’s triangle from the non-pathological side into the target disc.

### Disc-FX

For Disc-FX, a guidewire was advanced through the puncture needle and a working cannula was introduced over the annulus. Manual discectomy was first performed with gripping forceps to remove herniated disc material. A bipolar radiofrequency electrode (Turbo mode) was then used to ablate the nucleus pulposus in several short bursts, followed by annular modulation in Hemo mode with stepwise rotation of the electrode. The disc space was repeatedly irrigated with saline through the working channel.

### L’DISQ

For L’DISQ, a cannula and navigable flexible electrode were advanced into the disc toward the herniated lesion. A short-burst electrical stimulation test was performed to ensure that the electrode was located in the painful area and not too close to the nerve root; if radiating pain or muscle contraction occurred, the electrode was repositioned. Disc tissue was then ablated using brief bursts of radiofrequency energy with intermittent repositioning, for a total ablation time of approximately 200–400 s. The coagulation mode was not used in this cohort. Periprocedural adverse events were prospectively recorded, and all patients underwent routine neurological examination and monitoring after the procedure. All patients were discharged within 1–2 days.

### Clinical outcomes and follow-up

Pain intensity was assessed using the 11-point Numerical Rating Scale (NRS; 0 = no pain, 10 = worst pain), and health-related quality of life (QOL) was evaluated using the EuroQOL 5-Dimension 5-Levels (EQ-5D-5 L) questionnaire, which covers mobility, self-care, usual activities, anxiety/depression, and pain/discomfort [9, 10]. At baseline and each follow-up visit (1 week, 1 month, 3 months, and 6 months postoperatively), NRS and EQ-5D-5 L were recorded by the same two attending pain clinic surgeons who performed the procedures. Both surgeons were aware of whether the patient had undergone Disc-FX or L’DISQ. Demographic and clinical characteristics were obtained from clinical records.

The primary outcome was the between-group difference in change in NRS score from baseline to 6 months postoperatively. Key secondary outcomes were the between-group difference in 6-month change in EQ-5D-5 L and the proportions of patients achieving the prespecified minimal clinically important difference (MCID) thresholds for NRS and EQ-5D-5 L at 6 months. Additional secondary/exploratory outcomes included (1) trajectories of NRS and EQ-5D-5 L at all follow-up time points, (2) achievement of the NRS MCID after provocative discography, (3) reduction in tramadol consumption at 6 months, and (4) the need for additional pain procedures (e.g. pulsed radiofrequency) or spinal surgery within 6 months of PDD.

The MCID was defined as the smallest change in outcome that patients perceive as important [11]. Based on the published literature [11, 12], we defined MCID as a reduction of ≥ 2 points in NRS and an increase of ≥ 0.08 points in EQ-5D-5 L. Use of other oral analgesic and neuropathic medications (e.g. nonsteroidal anti-inflammatory drugs, pregabalin, mirogabalin, duloxetine, tramadol) were left to the discretion of referring physicians and were not standardized by protocol. Baseline and 6-month medication records for these agents were available for all patients and were summarized descriptively as a post hoc exploratory analysis (Table 4).

### Statistical analysis

All analyses were performed using JMP Pro 17 (SAS Institute Inc., Cary, NC, USA). Continuous variables are presented as mean ± standard deviation (SD), and categorical variables as counts and percentages. Between-group comparisons of baseline characteristics used t tests (or Welch’s t test when variance heterogeneity was suspected) for continuous variables and Fisher’s exact or χ² tests for categorical variables.

Within-group changes from baseline in NRS and EQ-5D-5 L were assessed using the Wilcoxon signed-rank test, and between-group comparisons at each time point and for 6-month change scores were performed using the Wilcoxon rank-sum test (Mann–Whitney U test). For the primary and key secondary continuous outcomes, we estimated between-group mean differences in 6-month changes in NRS and EQ-5D-5 L, their 95% confidence intervals, and standardized effect sizes (Hedges’ g). For binary outcomes (MCID attainment), we calculated risk differences (Newcombe hybrid-score 95% confidence intervals), risk ratios (score-based 95% confidence intervals), and odds ratios (exact 95% confidence intervals), and used Fisher’s exact test for hypothesis testing.

In exploratory multivariable analyses, we used linear regression (analysis of covariance) models for 6-month changes in NRS and EQ-5D-5 L and logistic regression models for MCID attainment, including treatment group, positive provocative discography, and relevant baseline covariates (e.g. baseline NRS or EQ-5D-5 L, age, sex, and body mass index). All tests were two-sided, and *p* values < 0.05 were considered statistically significant. All available data were analyzed; missing follow-up NRS and EQ-5D-5 L values were imputed using the last observation carried forward, and patients without baseline NRS or EQ-5D-5 L data were excluded from the corresponding analyses.

## Results

### Basic characteristics of the participants

**Fig. 1:**
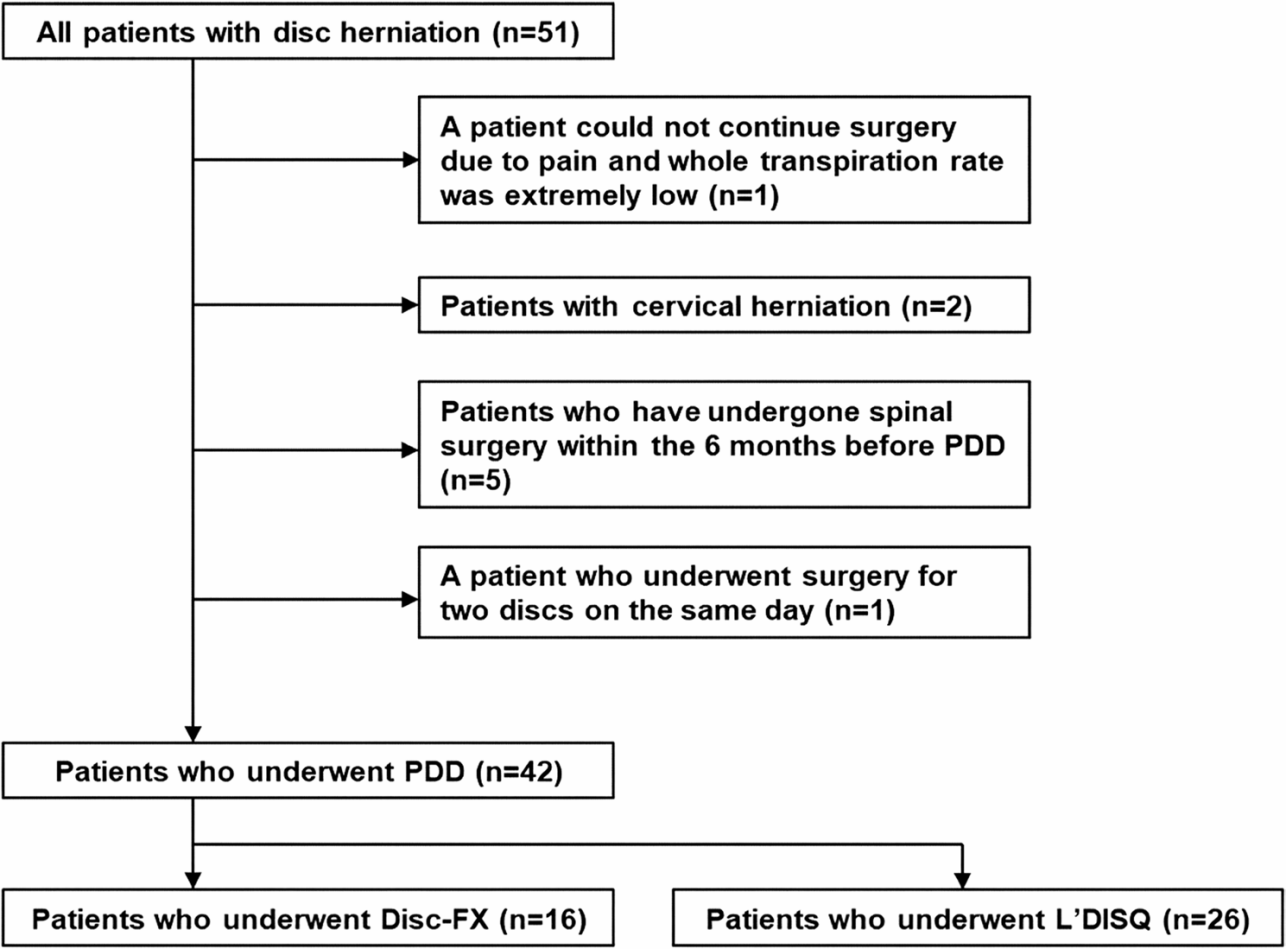
Flowchart of patient inclusion

The flowchart of patient inclusion is presented in Fig. 1. Of the 51 patients, 42 were included in this study. Demographic data are summarized in Table 1. Positive provocative discography differed significantly between the groups (Disc-FX 8/16 [50.0%] vs. L’DISQ 23/26 [88.5%], *p* = 0.011). Baseline NRS was higher in the Disc-FX group (*p* < 0.05), whereas baseline EQ-5D-5 L did not differ significantly.

**Table 1:** Basic characteristics of overall patients

	Total		Disc-FX		L’DISQ		*P*-value
	(*n* = 42)	%	(*n* =16)	%	(*n* = 26)	%	
Age, years, mean (SD)	57 (17.8)		57.9 (18.7)		56.3 (17.5)		0.766
Women	21	50.0	7	43.8	14	53.8	0.751
Height, cm, mean (SD)	163.3 (10.3)		161.9 (8.9)		164.2 (11.2)		0.422
Weight, kg, mean (SD)	67.8 (15.0)		66.6 (17.5)		68.5 (13.5)		0.560
BMI, kg/m^2^, mean (SD)	25.3 (4.8)		25.3 (5.6)		25.4 (4.4)		0.796
Diabetes mellitus	4	9.5	0	0	4	15.4	0.280
Smoking							0.644
now	8	19.0	2	12.5	6	23.1	
past	6	14.3	3	18.8	3	11.5	
never	28	66.7	11	68.8	17	65.4	
Intervertebral disc level							0.142
L2/3	3	7.1	2	12.5	1	3.8	
L3/4	4	9.5	2	12.5	2	7.7	
L4/5	13	31.0	7	43.8	6	23.1	
L5/S1	22	52.4	5	31.3	17	65.4	
Pulsed radiofrequency before PDD	21	50.0	9	56.3	12	46.2	0.525
Pulsed radiofrequency after PDD	21	50.0	8	50.0	13	50.0	1.000
Other surgery in 6 months	5	14.3	1	6.3	4	15.4	0.375
Pfirrmann classification						0.0	0.206
2	1	2.4	1	6.3	0	0.0	
3	4	9.5	1	6.3	3	11.5	
4	33	78.6	14	87.5	19	73.1	
5	4	9.5	0	0.0	4	15.4	
MacNab classification							
protrusion	1	2.4	0	0.0	1	3.8	0.490
subligamentous extrusion	27	64.3	10	62.5	17	65.4	0.935
transligamentous extrusion	12	28.6	5	31.3	7	26.9	0.881
unknown	2	4.8	1	6.3	1	3.8	
Cephalad/caudal migration	3	7.1	2	12.5	1	3.8	0.290
Broadly herniated disc	35	83.3	14	87.5	21	80.8	0.747
High intensity zone	13	31.0	4	25.0	9	34.6	0.513
Spondylolisthesis	1	2.4	0	0.0	1	3.8	0.418
Thecal sac compression	23	54.8	8	50.0	15	57.7	0.627
Central stenosis	5	11.9	3	18.8	2	7.7	0.283
Foraminal stenosis	31	73.8	11	68.8	20	76.9	0.559
Positive provocative discography	31	73.8	8	50.0	23	88.5	0.011

*P* values: The Disc-FX group vs. the L’DISQ group

### NRS scores

**Fig. 2:**
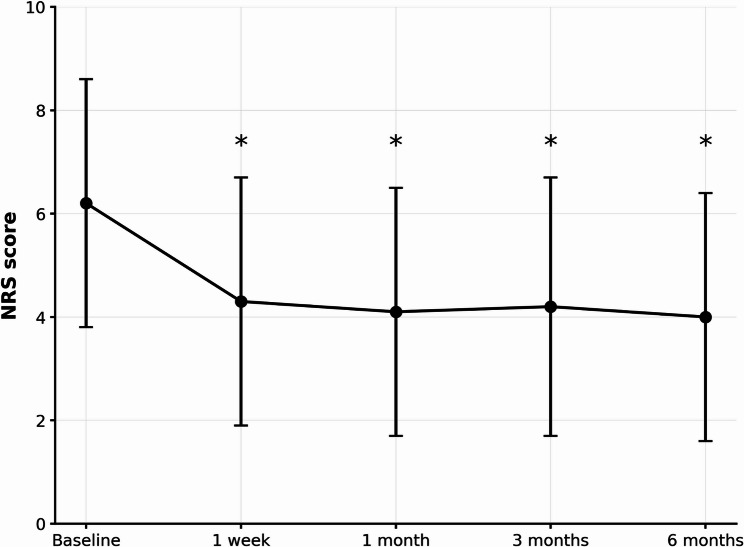
Changes in the overall mean numeric rating scale (NRS) score for all patients over the 6-month follow-up. The mean ± SD NRS scores at baseline, 1 week, 1 month, 3 months, and 6 months were 6.21 ± 2.27, 4.33 ± 2.45, 4.12 ± 2.40, 4.19 ± 2.60, and 4.10 ± 2.29, respectively. * indicates significant differences compared to baseline (*p* < 0.05; Wilcoxon signed-rank test). Error bars indicate standard deviation

**Fig. 3:**
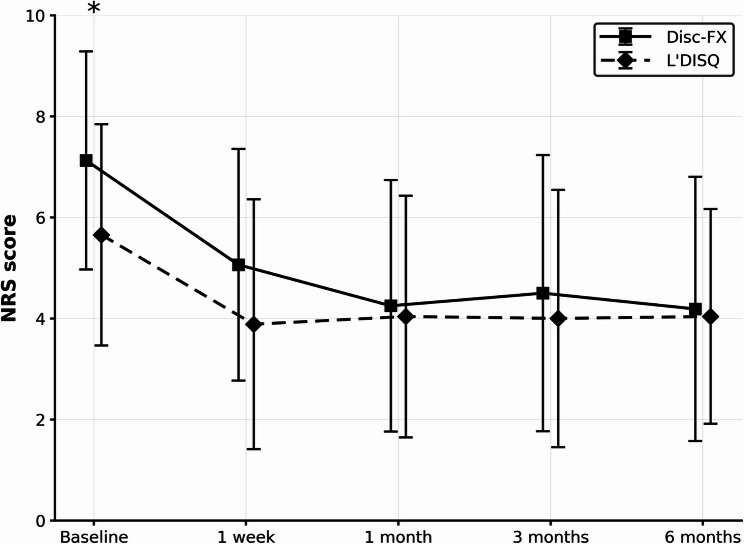
Comparison of the mean NRS pain scores at each time point between the Disc-FX and L’DISQ groups. At baseline, the Disc-FX group had a significantly higher mean NRS than the L’DISQ group (7.13 ± 2.16 vs. 5.65 ± 2.19; *p* < 0.05), whereas no significant between-group differences were observed at any postoperative time point. * indicates *p* < 0.05 for between-group comparisons. Error bars indicate standard deviation. At 1 week, 1 month, 3 months, and 6 months postoperatively, the mean NRS for Disc-FX vs. L’DISQ were 5.06 ± 2.29 vs. 3.88 ± 2.47, 4.25 ± 2.49 vs. 4.04 ± 2.39, 4.50 ± 2.73 vs. 4.00 ± 2.55, and 4.19 ± 2.61 vs. 4.04 ± 2.13, respectively

In both treatment groups, NRS pain scores decreased substantially from baseline and remained significantly lower at all postoperative follow-ups (all *p* < 0.05 vs. baseline; Fig. 2). At baseline, the Disc-FX group reported significantly higher pain intensity than the L’DISQ group (mean NRS 7.13 ± 2.16 vs. 5.65 ± 2.19; *p* < 0.05), but postoperative NRS trajectories were similar and there were no significant between-group differences in NRS at any follow-up visit (Fig. 3). At 6 months, mean NRS improvement was 2.94 ± 2.69 in the Disc-FX group and 1.62 ± 2.42 in the L’DISQ group, corresponding to a between-group mean difference of 1.32 points (Disc-FX minus L’DISQ; 95% confidence interval [CI] − 0.36 to 3.01; Hedges’ g 0.51, 95% CI − 0.12 to 1.15; *p* = 0.0969). Overall, 24/42 patients (57.1%) achieved the prespecified MCID for the NRS score at 6 months; 11/16 (68.8%) in the Disc-FX group and 13/26 (50.0%) in the L’DISQ group achieved the MCID (risk difference + 18.8% points, 95% CI − 11.5 to 43.5; risk ratio 1.38, 95% CI 0.79–2.32; odds ratio 2.20, 95% CI 0.60–8.13; two-sided Fisher’s exact test *p* = 0.338; Table 2). Thus, both procedures yielded clinically meaningful pain relief, and the primary outcome (between-group difference in 6-month NRS change) did not differ significantly between Disc-FX and L’DISQ in this retrospective pilot cohort. Consistent with this, the change in NRS immediately after provocative discography and the proportion of patients achieving the NRS MCID after discography did not differ significantly between the two groups (Table 3).

**Table 2:** Overall success rate and success rates for the Disc-FX and L’DISQ groups in achieving the MCID for both NRS and EQ-5D-5 L scores at 6 months postoperatively

	Total		Disc-FX		L’DISQ		*P*-value
	*n*	%	*n*	%	*n*	%	
NRS reached MCID in 6 months	24/42	57.1	11/16	68.8	13/26	50.0	0.338
EQ-5D-5 L reached MCID in 6 months	23/38	60.5	10/12	83.3	13/26	50.0	0.077

At 6 months, the absolute risk difference (Disc-FX minus L’DISQ) for achieving the NRS MCID was +18.8 percentage points (95% CI −11.5 to 43.5; risk ratio 1.38, 95% CI 0.79–2.32; odds ratio 2.20, 95% CI 0.60–8.13). For EQ-5D-5L MCID, the absolute risk difference was +33.3 percentage points (95% CI −0.04 to 54.9; risk ratio 1.67, 95% CI 0.99–2.72; odds ratio 5.00, 95% CI 0.91–27.4; two-sided Fisher’s exact test *p* = 0.077).

For EQ-5D-5 L, four patients with missing baseline EQ-5D-5 L data were excluded (*n* = 38)

*NRS* numerical rating scale, *MCID* minimal clinically important difference

**Table 3:** Amount of change in NRS score after discography and the number of people whose NRS score achieved the MCID after discography

	Total	Disc-FX	L’DISQ	*P*-value
	(*n* = 41)	(*n* = 15)	(*n* = 26)	
The amount of change in NRS score after discography (SD)	-1.9 (2.1)	-2.7 (2.6)	-1.4 (1.7)	0.095
NRS reached MCID after discography, n/N (%)	17(41.5)	9(60.0)	8(30.8)	0.102

*SD* standard deviation, *NRS* numerical rating scale, *MCID* minimal clinically important difference

Analyses are based on available data; one Disc-FX case had a missing value for the NRS change after discography and was excluded

### EQ-5D-5 L score

**Fig. 4:**
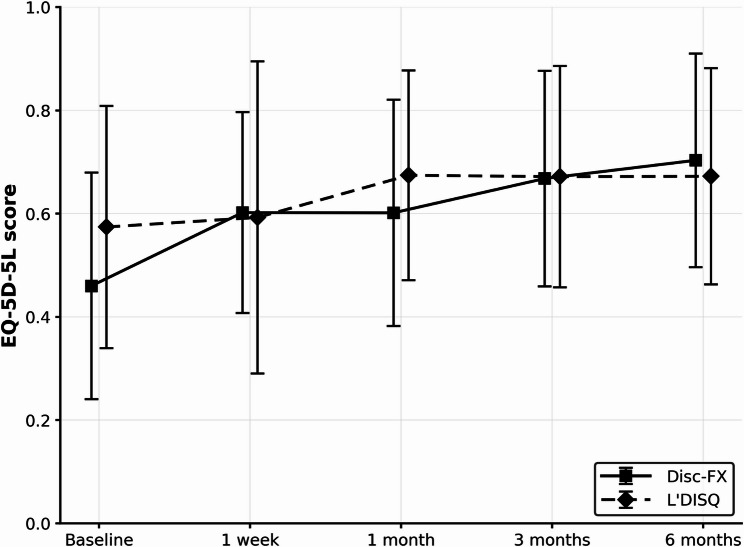
Comparison of the mean EQ-5D-5 L quality-of-life scores at each time point between the Disc-FX and L’DISQ groups (analysis *n* = 38; Disc-FX *n* = 12, L’DISQ *n* = 26; baseline EQ-5D-5 L data available). The mean ± SD EQ-5D-5 L values for the Disc-FX vs. L’DISQ groups at baseline, 1 week, 1 month, 3 months, and 6 months were 0.46 ± 0.22 vs. 0.57 ± 0.23, 0.60 ± 0.20 vs. 0.59 ± 0.30, 0.61 ± 0.21 vs. 0.67 ± 0.20, 0.65 ± 0.22 vs. 0.67 ± 0.21, and 0.69 ± 0.23 vs. 0.67 ± 0.21, respectively. No significant differences were observed between the two groups at any timepoint. Error bars indicate standard deviation

**Fig. 5:**
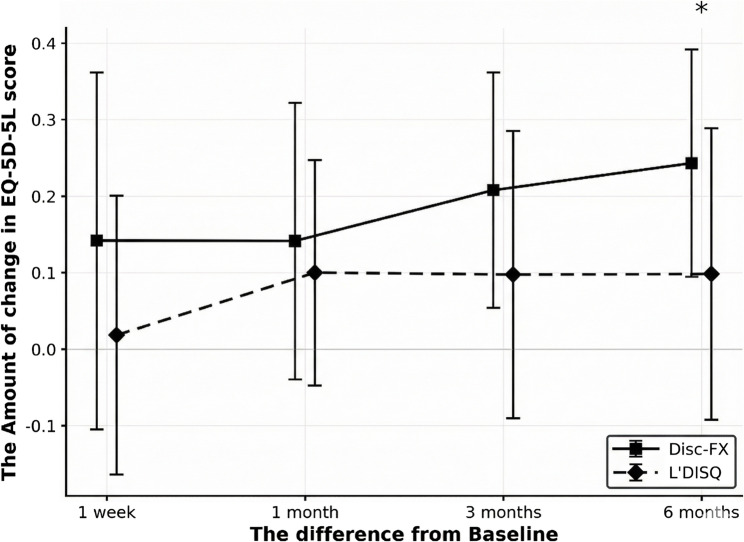
Amount of change from baseline in EQ-5D-5 L score at 1 week, 1 month, 3 months, and 6 months postoperatively for the Disc-FX and L’DISQ groups. The mean ± SD changes in EQ-5D-5 L from baseline for Disc-FX vs. L’DISQ were + 0.14 ± 0.26 vs. + 0.02 ± 0.19 at 1 week, + 0.15 ± 0.19 vs. + 0.10 ± 0.15 at 1 month, + 0.19 ± 0.16 vs. + 0.10 ± 0.19 at 3 months, and + 0.23 ± 0.16 vs. + 0.10 ± 0.19 at 6 months. * indicates a significant difference between the Disc-FX and L’DISQ groups at 6 months (*p* = 0.0183; Wilcoxon rank-sum test). Error bars indicate standard deviation

Among the 38 patients with baseline EQ-5D-5 L data (Disc-FX *n* = 12, L’DISQ *n* = 26), mean scores improved over time in both groups (Fig. 4). There were no significant between-group differences in mean EQ-5D-5 L values at baseline, 1 week, 1 month, or 3 months postoperatively. At 6 months, however, the Disc-FX group showed greater improvement than the L’DISQ group: mean change from baseline 0.23 ± 0.16 vs. 0.10 ± 0.19, respectively (between-group mean difference 0.134; 95% confidence interval [CI] 0.013–0.255; *p* = 0.0183; Hedges’ g 0.71; Fig. 5). At 6 months, 23/38 patients (60.5%) achieved the EQ-5D-5 L MCID; 10/12 (83.3%) in the Disc-FX group and 13/26 (50.0%) in the L’DISQ group achieved the MCID (absolute risk difference 33.3% points; 95% CI − 0.04–54.9; risk ratio 1.67; 95% CI 0.99–2.72; odds ratio 5.00; 95% CI 0.91–27.4; two-sided Fisher’s exact test *p* = 0.077; Table 2).

In exploratory multivariable linear regression adjusting for baseline scores and positive provocative discography, the adjusted between-group differences in 6-month change remained small and imprecise: 0.1 points for NRS (Disc-FX minus L’DISQ; 95% CI − 1.5 to 1.7; *p* = 0.91) and 0.04 for EQ-5D-5 L (95% CI − 0.08 to 0.16; *p* = 0.55). Positive provocative discography was associated with a modestly smaller EQ-5D-5 L improvement (β − 0.14; 95% CI − 0.27 to − 0.01; *p* = 0.045). In exploratory logistic regression, the adjusted odds ratios (Disc-FX vs. L’DISQ) for achieving the MCID at 6 months were 1.73 (95% CI 0.42–7.21; *p* = 0.45) for NRS and 4.25 (95% CI 0.70–25.95; *p* = 0.12) for EQ-5D-5 L; positive provocative discography was not significantly associated with MCID achievement in either model (all *p* > 0.40).

### Postoperative analgesia and additional pain procedure

Overall, 10 patients (23.8%) were able to reduce their tramadol dosage at 6 months postoperative follow-up compared to the preoperative baseline: four patients (25.0%) in the Disc-FX group and six patients (23.1%) in the L’DISQ group. No significant between-group differences were observed.

In an exploratory descriptive analysis of concomitant oral medications, any neuropathic agent (pregabalin, mirogabalin, or duloxetine) was used preoperatively by 10/16 patients (62.5%) in the Disc-FX group and 15/26 patients (57.7%) in the L’DISQ group, decreasing to 5/16 (31.3%) and 9/26 (34.6%), respectively, at 6 months When NSAIDs and acetaminophen were considered together as any oral analgesic, the proportions were 12/16 (75.0%) versus 14/26 (53.8%) at baseline and 5/16 (31.3%) versus 12/26 (46.2%) at 6 months in the Disc-FX and L’DISQ groups, respectively. Details of concomitant tramadol, NSAIDs, acetaminophen, pregabalin, mirogabalin, and duloxetine at baseline and 6 months are summarized in Table 4.

**Table 4:** Concomitant use of Tramadol and other oral analgesic and neuropathic medications at baseline and 6 months postoperatively in the Disc-FX and L’DISQ groups

	Disc-FX baseline, *n* (%)	Disc-FX 6 months, *n* (%)	L’DISQ baseline, *n* (%)	L’DISQ 6 months, *n* (%)
Tramadol	5 (31.3)	3 (18.8)	13 (50.0)	8 (30.8)
NSAIDs	9 (56.3)	5 (31.3)	8 (30.8)	8 (30.8)
Acetaminophen	6 (37.5)	2 (12.5)	10 (38.5)	7 (26.9)
Pregabalin	6 (37.5)	3 (18.8)	5 (19.2)	1 (3.8)
Mirogabalin	4 (25.0)	2 (12.5)	6 (23.1)	4 (15.4)
Duloxetine	2 (12.5)	1 (6.3)	6 (23.1)	7 (26.9)

The number of patients who received high-frequency pulse therapy within 1 month of surgery as an additional treatment was 16 (5 in the Disc-FX group and 11 in the L’DISQ group), and no significant between-group difference was detected (*p* = 0.23). Five patients (one in the Disc-FX group and four in the L’DISQ group) underwent postoperative spinal surgery at the same disc level where PDD had been performed, within 6 months postoperatively. There was no significant difference between the groups (*p* = 0.375). In the Disc-FX group, one patient underwent laminectomy, whereas in the L’DISQ group, two patients underwent posterior discectomy, one underwent spinal fusion surgery, and one underwent laminoplasty.

## Discussion

In this retrospective pilot comparative study, both Disc-FX and L’DISQ were associated with significant improvements in NRS pain scores over 6 months, and the primary outcome—the between-group difference in 6-month NRS change—did not differ significantly between the two devices. These findings indicate that both minimally invasive procedures provided comparable short-term pain relief for patients with lumbar disc herniation. As key secondary outcomes, the Disc-FX group showed greater improvement in EQ-5D-5 L and a higher EQ-5D-5 L MCID attainment rate at 6 months than the L’DISQ group; however, given the small sample size, baseline imbalances (particularly in provocative discography), and the retrospective design, these QOL-related differences should be interpreted as exploratory and hypothesis-generating rather than definitive evidence of Disc-FX superiority.

The pain index associated with discogenic pain decreased after treatment with both Disc-FX and L’DISQ, and our findings are consistent with those of previous studies. A systematic review of 15 studies involving 570 patients using Disc-FX was conducted. The findings indicated that over 2 to 24 months, the visual analog scale (VAS) score decreased from 7.22 to 1.81, and the average NRS score decreased from 6.98 to 3.90 [5]. In this study, the NRS score of the 16 participants in the Disc-FX group decreased from 7.12 to 4.19 over the 6-month period following treatment. In contrast, Lee et al. reported that among 27 patients with herniated discs, the VAS score decreased from 7.08 before treatment to 1.08 at 24 weeks post-treatment, using L’DISQ [13]. Furthermore, in 2015, they observed a reduction in VAS score from 7.55 to 3.60 at 48 weeks after the L’DISQ treatment in 20 patients with discogenic low back pain [14]. Our 6-month findings in the Disc-FX group appear directionally consistent with the early phase of the long-term Disc-FX outcomes [15] reported in the literature, although they should be regarded as preliminary and not as definitive evidence of long-term superiority.

This study demonstrated a significant between-group difference in the 6-month change in EQ-5D-5 L favouring Disc-FX, and the Disc-FX group also had a numerically higher proportion of patients achieving the EQ-5D-5 L MCID at 6 months (83.3% vs. 50.0%); however, this MCID difference did not reach conventional statistical significance and should be interpreted as an exploratory finding. Previous studies have shown that both the Disc-FX and L’DISQ are effective in reducing the Oswestry disability index (ODI), a measurement of disability in patients with low back pain. In a systematic review of Disc-FX by Lin et al., the mean ODI score decreased from 35.37 to 14.66 points from preoperative to the final follow-up [5]. In L’DISQ, the mean ODI score from preoperative to 12 months postoperative follow-up decreased significantly from 32.46 to 20.48 [16]. Furthermore, in a prospective cohort study, the mean ODI 2 years after treatment also decreased from 50.9 to 20.3 [17].

The between-group differences observed in EQ-5D-5 L at 6 months may partly reflect differences in the extent and mode of disc tissue removal achieved by the two devices. Previous reports have shown that Disc-FX typically removes approximately 0.8 g of disc tissue through bipolar nucleus ablation, whereas L’DISQ plasma ablation removes around 1.35 g of nucleus pulposus under experimental conditions [17, 18]. In addition, Disc-FX allows manual removal of disc material using forceps, although the actual volume removed may vary depending on disc hydration and disc morphology. It is therefore conceivable that differences in the amount of nucleus removed could be associated with the degree of improvement in patient-reported QOL. Furthermore, Disc-FX was the only device in our cohort for which we used a coagulation mode corresponding to annular thermal modification. In theory, annular thermal modification may tighten the annulus via collagen shrinkage, potentially narrowing annular fissures and providing a partial, functional sealing effect. Annular closure devices designed to seal annular defects have been reported to improve postoperative pain, patient satisfaction, and activities of daily living in other settings [19]. Although we did not evaluate structural or imaging outcomes such as disc height, nucleus volume, annular integrity, or radiological reherniation in the present study, these device-specific features may represent one plausible, but still hypothetical, explanation for the better EQ-5D-5 L response observed with Disc-FX.

When selecting a treatment, multiple indicators should be taken into account, including QOL scales. This study indicated that the number of additional treatments with pulsed radiofrequency or spinal surgery were similar in the two group. These additional interventions may reflect persistent or recurrent symptoms (including possible reherniation); however, radiological recurrence was not systematically assessed in our cohort, so the underlying mechanism cannot be confirmed, thus the results should be interpreted with caution. The risk of reoperation is heightened when there is a defect in the annulus following disc surgery [20]. In patients where re-prolapse is confirmed, an increase in NRS scores is observed even in the early postoperative period [21]. Furthermore, a previous study demonstrated that 15% of reoperations were performed within 1 month of the first operation [22]. Additional pulsed radiofrequency within 1 month could indicate early symptom persistence or recurrence; however, attributing this pattern specifically to re-protrusion remains speculative without imaging confirmation. Compared with some conventional decompression techniques, Disc-FX incorporates annuloplasty and annular thermal modulation; these adjuncts have been proposed as strategies that might reduce recurrent herniation, but this hypothesis was not evaluated in the present study.

However, in our series we did not perform annular coagulation with L’DISQ and we did not systematically assess radiological reherniation. Therefore, any inference that Disc-FX or L’DISQ reduces reherniation through annular modification should be regarded as speculative. In this exploratory cohort, the small cannula diameters of both devices (3.4 mm for Disc-FX and 2.0 mm for L’DISQ) and the overall minimally invasive nature of the procedures may also have contributed to the low frequency of additional interventions.

### Limitations

This study has several important limitations. First, it was a single-center retrospective pilot cohort with a small sample size and without a formal a priori sample size calculation. Treatment allocation to Disc-FX or L’DISQ was determined in routine clinical practice and was strongly associated with provocative discography findings; positive discography was substantially more frequent in the L’DISQ group than in the Disc-FX group. This imbalance indicates selection bias and raises the possibility of residual confounding by baseline disease severity, symptom duration, or pain mechanisms, even though our exploratory multivariable analyses adjusting for provocative discography and other baseline factors did not materially change the direction of the between-group differences. In addition, the main clinical outcomes were patient-reported measures of pain (NRS) and health-related quality of life (EQ-5D-5 L), assessed by the same two treating pain clinic surgeons who were aware of the allocated procedure; although this may have reduced inter-observer variability, the lack of blinded outcome assessment introduces a risk of observer or information bias. Second, because both NRS and EQ-5D-5 L have fixed upper and lower bounds, our findings are susceptible to ceiling effects, especially in patients with more severe baseline symptoms for whom larger numerical improvements are mathematically possible; this may have exaggerated apparent treatment effects and masked improvements in patients with milder baseline impairment [23]. Concomitant use and dosing of tramadol and other oral analgesic or neuropathic medications were not standardized by protocol and could only be summarized retrospectively in a descriptive manner, so confounding by differences in medication patterns between the Disc-FX and L’DISQ groups cannot be excluded. We also did not collect structural or imaging endpoints such as disc height, herniation volume, annular defect morphology, or radiological reherniation, and the clinical follow-up was limited to 6 months. Consequently, the structural mechanisms and long-term durability of the between-group difference in EQ-5D-5 L remain uncertain. Taken together, these limitations suggest that the observed between-group differences, including the apparent EQ-5D-5 L advantage of Disc-FX, should be interpreted as exploratory rather than definitive. Future adequately powered, prospective multicenter studies, ideally randomized controlled trials comparing Disc-FX and L’DISQ with standardized MRI-based and clinical assessments, are required to confirm the reproducibility and clinical significance of these findings.

## Conclusions

Both Disc-FX and L’DISQ provided comparable pain reduction, as reflected in similar 6-month changes in NRS, which was the primary outcome of this retrospective pilot study. Disc-FX showed greater improvement in EQ-5D-5 L and a higher EQ-5D-5 L MCID attainment rate at 6 months than L’DISQ, suggesting a possible advantage in health-related QOL. However, these secondary findings arise from a small single-centre retrospective cohort should therefore be regarded as hypothesis-generating rather than definitive evidence of superiority. Larger prospective comparative studies are required to confirm whether the observed QOL differences are reproducible and clinically meaningful over the long term.

## Data Availability

The datasets generated and/or analyzed during the current study are not publicly available due to the inclusion of personally identifiable information, but are available from the corresponding author on reasonable request.

## Abbreviations

EQ-5D-5L: EuroQOL 5-Dimension 5-Levels
MCID: Minimal Clinically Important Difference
NRS: Numerical Rating Scale
ODI: Oswestry Disability Index
PDD: Percutaneous Disc Decompression
QOL: Quality of Life
SD: Standard Deviation
VAS: Visual Analog Scale
